# Discovery of CD3^+^CD19^+^ cells, a novel lymphocyte subset with a potential role in human immunodeficiency virus‐*Mycobacterium tuberculosis* coinfection, using mass cytometry

**DOI:** 10.1002/ctm2.681

**Published:** 2021-12-22

**Authors:** Qian Li, Jun Wang, Min Zhang, Yang Tang, Hongzhou Lu

**Affiliations:** ^1^ Department of Infectious Disease, The Third People's Hospital of Shenzhen, National Clinical Center for Infectious Disease the Second Affiliated Hospital of Southern University of Science and Techbology Shenzhen Guangdong China; ^2^ Bioinformatics and Computational Biophysics University of Duisburg‐Essen Essen Germany; ^3^ Department of Infectious Diseases Shanghai Public Health Clinical Center Shanghai China; ^4^ Clinical Laboratory The Fifth People's Hospital of Wuxi Jiangnan University Wuxi China

Dear Editor,

*Mycobacterium tuberculosis (Mtb)* is the leading cause of acquired immunodeficiency syndrome‐related death, with approximately 208 000 tuberculosis (TB) deaths occurring annually since 2019.^1^ Moreover, human immunodeficiency virus (HIV) co‐infection is a major risk factor for progression to active TB disease.^1^ The two pathogens act synergistically to accelerate the deterioration of the host immune system, posing a serious threat to public health. Therefore, questions have been raised regarding the phenotype of and changes in immunological function in individuals with HIV‐*Mtb* coinfection.

HIV infection dysregulates the *Mtb*‐specific T‐lymphocytic immune response.^2^ Moreover, elevated CD4^+^CD25^+^FoxP3^+^ with highly expressed programmed cell death 1 (PD1) has been reported to affect T‐cell functions in the pathogenesis of TB.^3^ However, the peripheral whole blood lymphocyte compartment and the possible pathologies of HIV‐*Mtb* coinfection have not been completely identified. Because of the large phenotypic diversity among lymphocyte populations, characterization of alterations across all immune cell subsets is difficult. Nevertheless, high‐dimensional, flow and mass cytometry, also known as CyTOF, could overcome the shortages and has been used to characterize immune populations in the field of infectious diseases and to predict biomarkers.^4^ In this context, we aimed to establish the immune cell landscape of peripheral blood mononuclear cells (PBMCs) in patients with HIV infection alone, *Mtb* infection alone, HIV‐*Mtb* coinfection and healthy controls (HCs) (Figure 1A).

**FIGURE 1:**
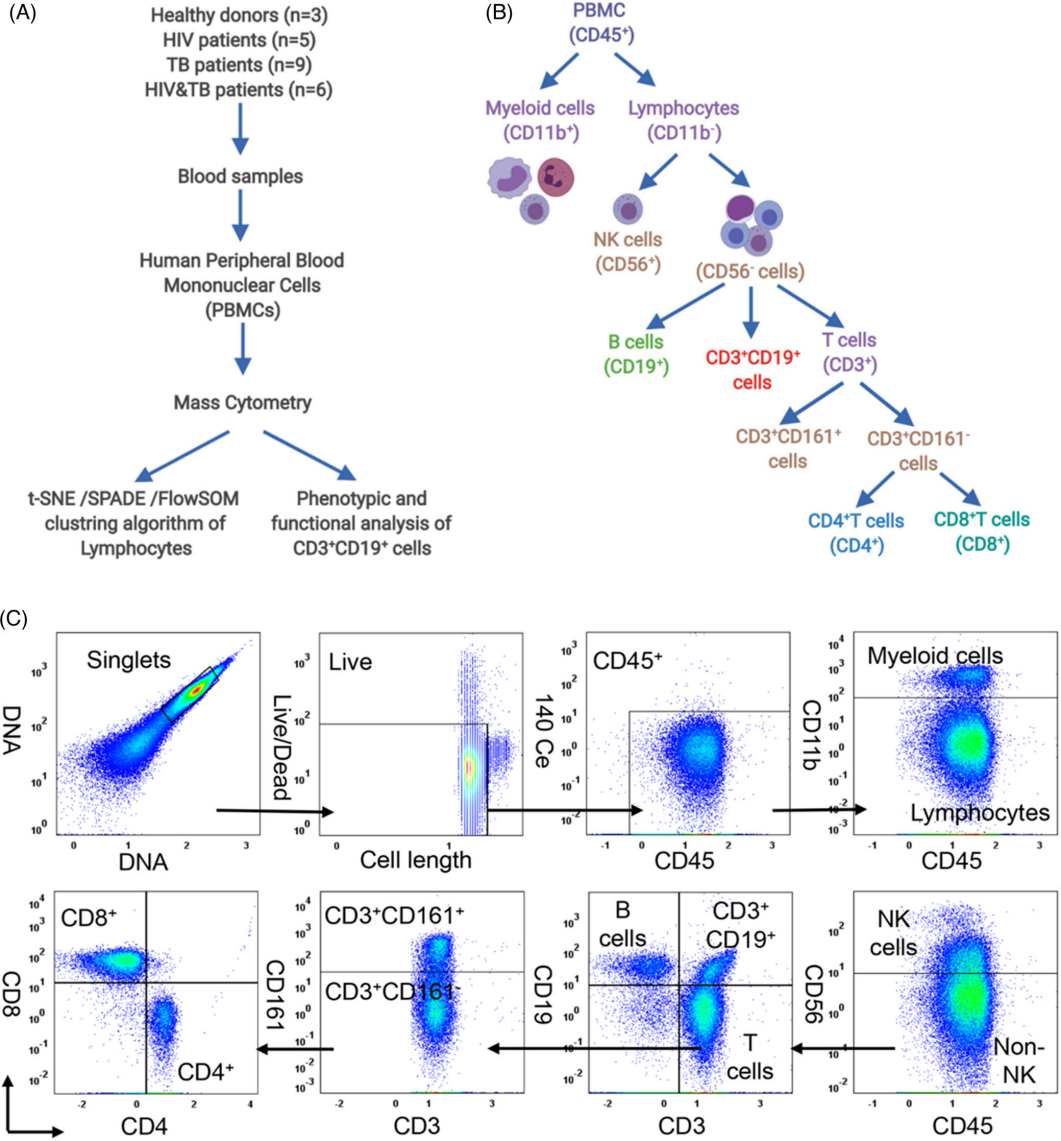
Schematic diagram of the experimental design and the gating strategy of time‐of‐flight cytometry (CyTOF). (A) Human peripheral blood specimens were collected from patients with human immunodeficiency virus (HIV) infection (*n* = 5), *Mycobacterium tuberculosis* (*Mtb*) infection (*n* = 9), HIV‐*Mtb* coinfection (*n* = 6) and healthy controls (HCs) (*n* = 3) at the Fifth People's Hospital of Wuxi and Shanghai Public Health Clinical Centre in accordance with the Declaration of Helsinki and with patients consent. All patients were aged between 20 and 60 years old. Cells were harvested and measured with the Helios system. Data were normalised in the software CyTOF using EQ Four Element Calibration Beads and analysed on Cytobank (https://www.cytobank.org/). Peripheral blood mononuclear cells (PBMCs) labelled with a metal‐tagged antibodies were divided into different immune populations. (B) A gating strategy was used to differentiate living intact single cells from debris, dead cells and cell aggregates. The immune cell population was gated with respective immune markers

Based on the combination of surface markers, PBMCs were manually gated as myeloid cells (CD45^+^CD11b^+^) and lymphocytes (CD45^+^CD11b^–^CD3^+^). Lymphocytes were further divided into nine major populations, including natural killer cells (CD56^+^), T cells (CD3^+^), B cells (CD19^+^), CD3^+^CD19^+^ cells, CD161^+^ cells, CD4^+^T cells, CD4^+^CD8^+^T cells, CD8^+^T cells and other cells (double‐negative lymphocytes) (Figure 1B,C). All samples were then subjected to viSNE and FlowSOM analysis,^5^ ^,^ ^6^ and a new lymphocyte subset, CD3^+^CD19^+^ cells, was characterized with high CD45, CD3 and CD19 expression; slight CD11b and CD56 expression; and barely CD4 and CD8 expression (Figure 2A). To our knowledge, CyTOF has not previously been used to identify CD3^+^CD19^+^ subsets in a complex disease scenario, including HIV infection, *Mtb* infection and HIV‐*Mtb* coinfection. The CD3^+^CD19^+^ cell counts were observed markedly reduced in patients with infection compared to those in HCs (Figure 2B,C).

**FIGURE 2:**
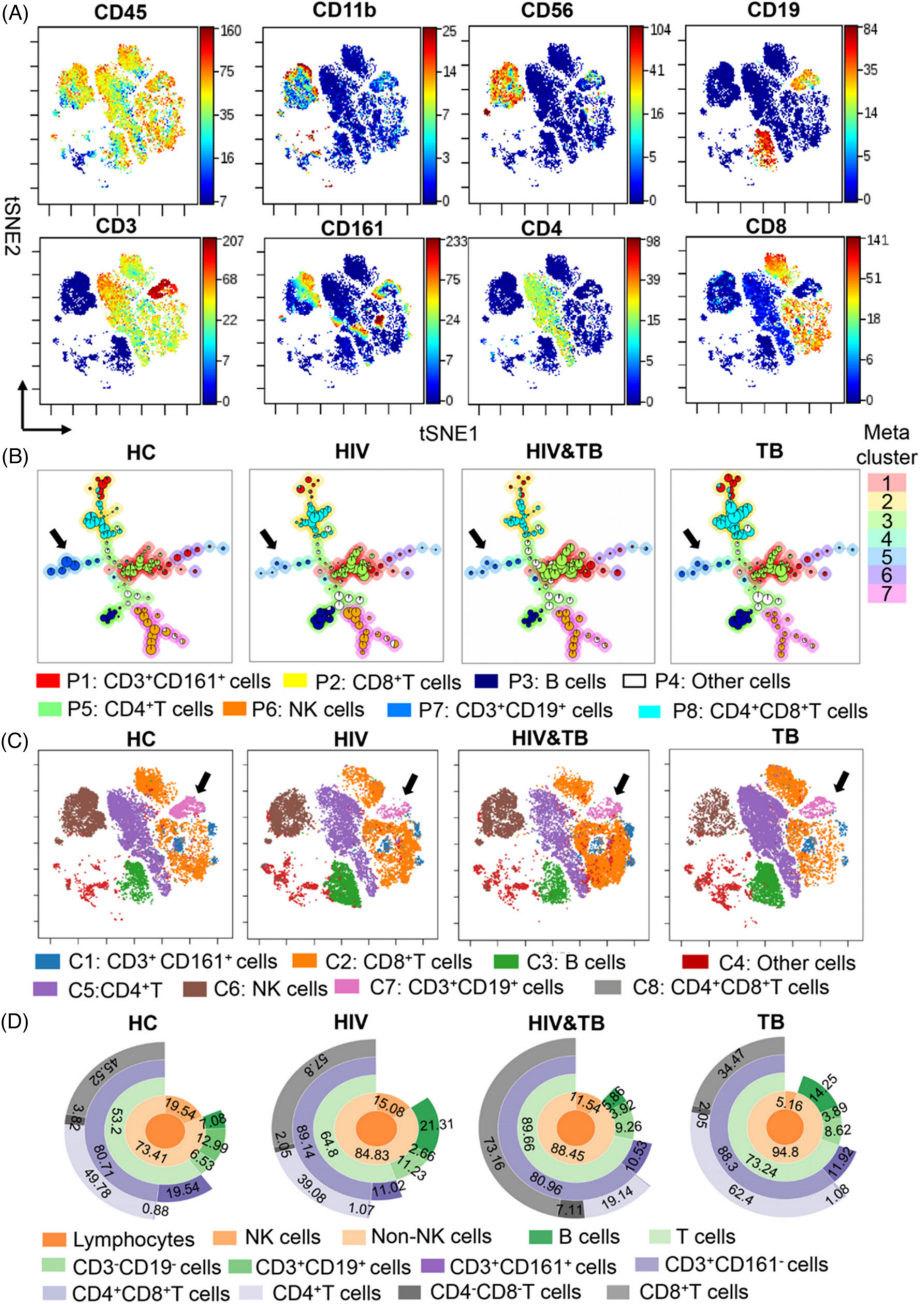
Validation of CD3^+^CD19^+^ cells and functional changes among patients with human immunodeficiency virus (HIV), *Mycobacterium tuberculosis* (*Mtb*) and HIV‐*Mtb* coinfection groups compared to healthy controls (HCs). (A) Eight surface markers (CD3, CD4, CD8, CD11b, CD19, CD45, CD56 and CD161) were used to construct a Visne map. (B and C) A FlowSOM and a Visne map of gated CD45^+^CD11b^–^CD56^–^CD3^+^ live single cells were used to depict the immune landscape. The black arrows in panels (B) and (C) indicate CD3^+^CD19^+^ subsets. (D) An abundance of lymphocyte cell subsets was generated using the sunburst method

The abundance of the 12 lymphocyte compartments is shown using a sunburst chart^7^ in Figure 2D. The frequency of CD3^+^CD19^+^ cells was .20‐, .30‐ and .30‐fold lower in the lymphocytes of individuals with HIV alone, HIV‐*Mtb* coinfection and *Mtb* alone, respectively, than in HCs, but was 1.47‐fold higher in HIV‐*Mtb* co‐infection group than that in the HIV group and 1.01‐fold higher than that in the TB group (Figure 3A). Eight major lymphocyte populations were characterized by using spanning‐tree progression analysis of density‐normalized events (SPADE),^7^ and a heatmap was shown to compare the level of expression of 41 immune functional markers in the lymphocyte populations of the three infected groups and HCs (Figure 3B,C). These markers were divided into four groups: (1) cluster differentiation markers that define distinct immune populations; (2) co‐stimulation markers including chemokine receptors, adhesion and activation molecules; (3) inhibitory markers; and (4) transcription factors. Expression of 20 immune functional markers by the novel CD3^+^CD19^+^ subset differed from that of cells expressing only CD3 or CD19 in the HCs (Figure 3B).

**FIGURE 3:**
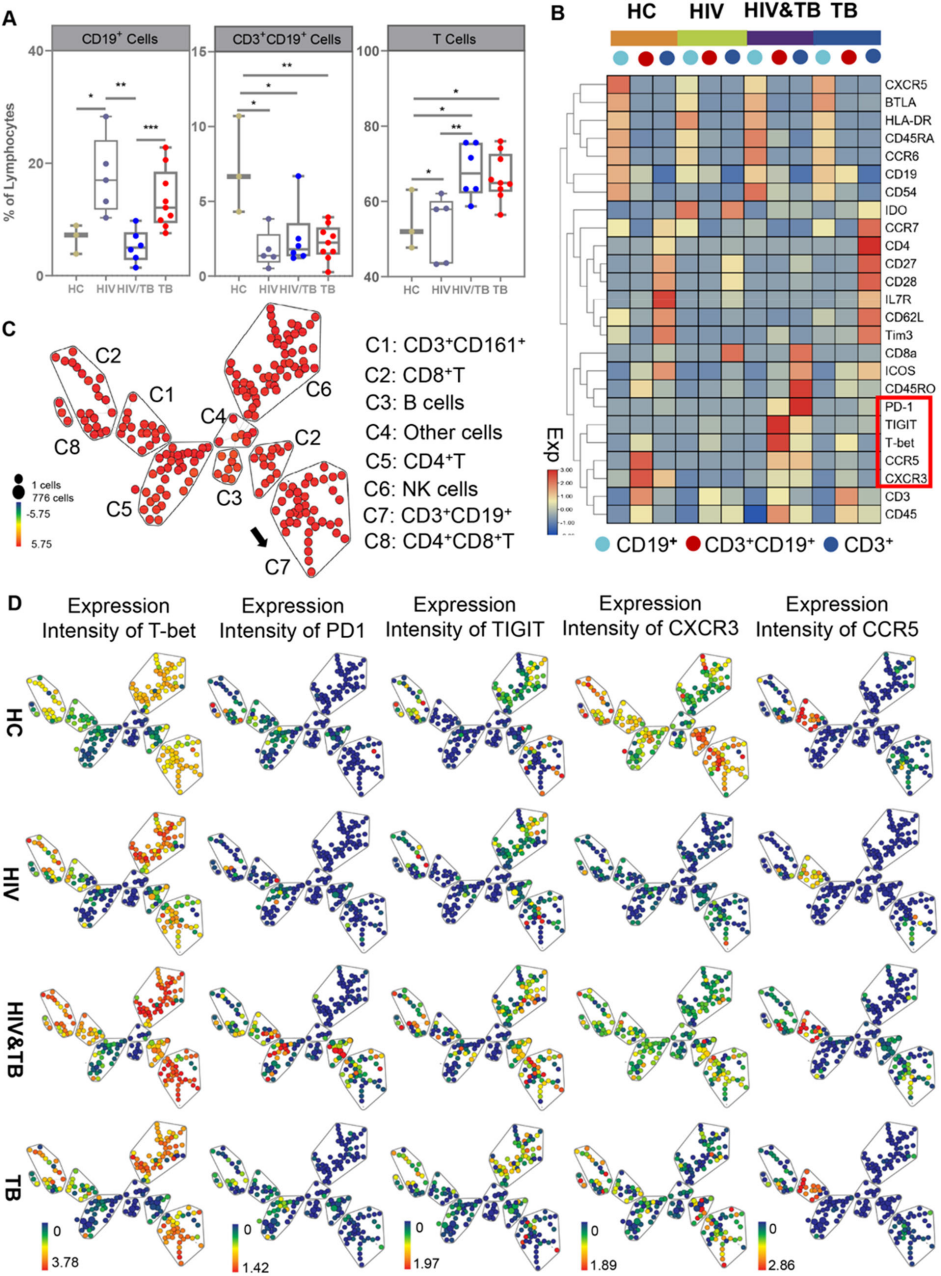
Characterization of the potential role of CD3^+^CD19^+^ cells and functional changes among the human immunodeficiency virus (HIV), *Mycobacterium tuberculosis* (*Mtb*) and HIV‐*Mtb* coinfection groups compared to healthy controls (HCs). (A) The frequency of CD19^+^ cells, CD3^+^CD19^+^ cells and CD3^+^ cells was further analysed using flow cytometry. Differences between each group were analysed using the Mann–Whitney *U* test. (B) A heat map representing median levels of marker expression for each population (CD3^+^ cells, CD3^+^CD19^+^ cells and CD19^+^ cells) was created using pheatmap. Differences in the levels of expression of 25 of 41 immune markers in samples from patients with HIV infection, *Mtb* infection, HIV‐*Mtb* coinfection and HCs were identified. (C) The SPADE algorithm was applied to gate CD45^+^CD11b^–^CD56^–^CD3^+^ from live and single cells to reveal the immune profile of eight major cell clusters for each cohort using the same surface markers in Figure 2A. (D) A SPADE plot was exhibited to show the median expression of T‐bet, PD1, TIGIT, CCR5 and CXCR3 in the eight lymphocyte subsets of HCs and patients with HIV infection, *Mtb* infection or HIV‐*Mtb* coinfection. Node size indicates the cell count, and colour gradient indicates the median value. Significant differences were indicated by *p < .05, **p < .01, ***p < .001

The cluster differentiation antigen CD45RA, the chemokine receptors CXCR5 and CCR6, the intercellular adhesion molecule CD54, the activation marker major histocompatibility complex, class II, DR (HLA‐DR) and the inhibitory molecule B‐ And T‐Lymphocyte attenuator (BTLA) were highly expressed in CD19^+^ cells. In addition, with the exception of HLA‐DR and indoleamine‐pyrrole 2,3‐dioxygenase (IDO), immune function markers including CXCR5, BTLA, CD45RA, CCR6 and CD54 were lower in the HIV group than in the other groups. Compared to the other groups, CCR6, CD45RA and CD54 were highly expressed on the CD19^+^ cells in the HIV‐*Mtb* co‐infection group. Compared to the HCs and the HIV‐*Mtb* coinfection group, HLA‐DR, CD45RA, CCR6 and CD54 were slightly expressed in the *Mtb* only group, but CXCR5 and BTLA were highly expressed in the *Mtb* group compared to the HIV‐*Mtb* coinfection group. The chemokine receptor CCR7, the cell adhesion molecule CD62L, the co‐simulation markers CD27, CD28, interleukin‐7 receptor (IL7R), inducible T‐cell costimulator (ICOS) and the co‐inhibitory molecules T cell immunoglobulin and mucin domain‐containing protein 3 (Tim3) were more highly expressed in CD3^+^ cells than the other two cell types. All of the markers except IL7R and ICOS were highly expressed in the *Mtb* group (Figure 3B).

Consistent with the decreased frequency of CD3^+^CD20^+^ cells in patients with HIV,^8^ CD3^+^CD19^+^ cells were also less abundant in the *Mtb* and HIV‐*Mtb* co‐infection groups than in the HCs. Moreover, a marked variation in the frequency of T and B cells was noted in the three infected groups, while a slight variation in the frequency of CD3^+^CD19^+^ cells was noted in the three infected groups. Notably, CCR5 and CXCR3 were highly expressed in CD3^+^CD19^+^ cells from HCs but were minimally expressed in the HIV group.^9^ CCR5 and CXCR3 expression was also lower in the HIV‐*Mtb* co‐infection group than in the HCs but was much higher than that in the other two groups (Figure 3D). The inhibitory molecules PD1 and T cell immunoreceptor with Ig and ITIM domains (TIGIT) were highly expressed in the CD3^+^CD19^+^ cells of the HIV‐*Mtb* coinfection group, indicating a phenotype involving greater suppression of the adaptive immune response. Notably, the transcription factor Tbx21 (T‐bet) was highly expressed as a result of stimulation by *Mtb* antigens, especially in the HIV‐*Mtb* coinfection group.^10^

## CONCLUSION

To our knowledge, this is the first study to use mass cytometry to reveal the adaptive immune landscape in samples from patients with HIV infection, *Mtb* infection and HIV‐*Mtb* coinfection and to discover a novel immune subset (CD3^+^CD19^+^ cells). The comprehensive analysis revealed the complex landscape of host immune response in response to pathogens including HIV, *Mtb* and HIV‐*Mtb* co‐infection. Refined analysis indicated that CD3^+^CD19^+^ cells in the HIV‐*Mtb* co‐infection group apparently had deceased expression of CCR5 and CXCR3 and increased expression of the inhibitory receptors PD1 and TIGIT. These findings may help to improve understanding of the immunopathogenesis of TB and HIV, and especially in individuals with HIV‐*Mtb* coinfection. Detailed study of the CD3^+^CD19^+^ subset and exploration of the underlying mechanisms for its involvement in HIV and *Mtb* infection and HIV‐*Mtb* coinfection may help to develop precise targeted treatments.

## CONFLICT OF INTEREST

The authors declare no competing interests.
